# A student‐led public education project on dermatology in skin of colour

**DOI:** 10.1002/ski2.166

**Published:** 2022-09-12

**Authors:** Pavel Loginovic, Natasha Syed, Alex M. Parker, Nel G. Williams, Navin Mukundu Nagesh

**Affiliations:** ^1^ University of Exeter Medical School College of Medicine and Health Exeter UK

## Abstract

Despite the UK's population rapidly diversifying, the representation of dermatological conditions in skin of colour in education, medical resources, and clinical practice is lagging. Furthermore, resources and advancements created by recent initiatives appear not to be communicated to the general public and are not integrated into medical curricula. In this perspective article, we share our experience from a public‐engagement campaign in South West England and propose that student‐led initiatives hold the potential to close the existing gap in diversity and racial equity in dermatology by communicating recent efforts within the medical field to the general public. We describe how student‐led initiatives allow medical students to advocate for diversity and equity within their institutions while delivering much‐needed education to ethnically minoritised communities.

## Graphical abstract

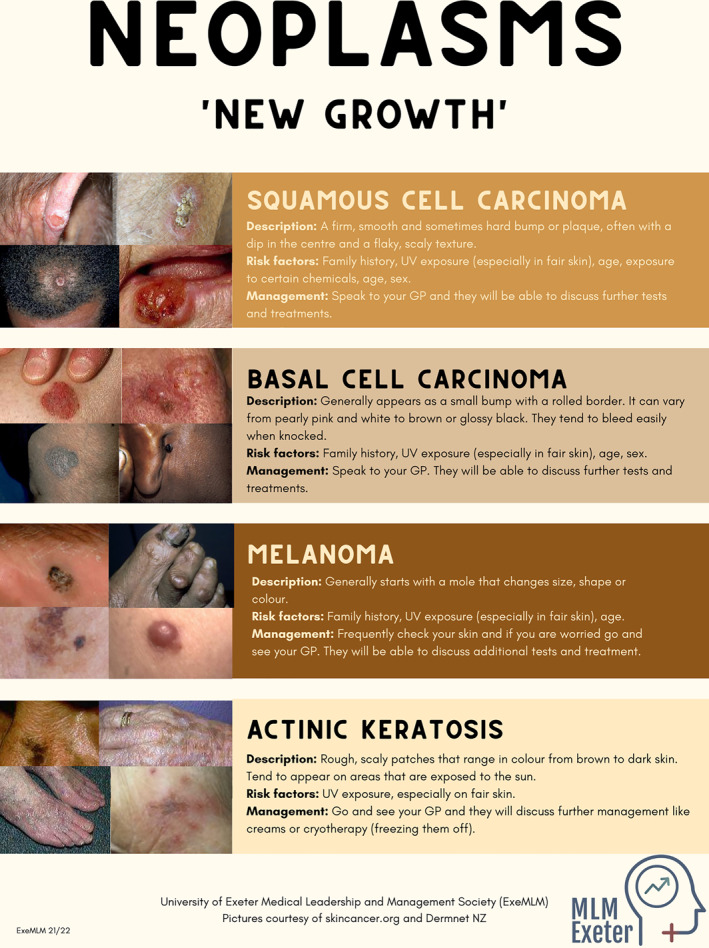

Despite the UK's population rapidly diversifying, the diversity in dermatological education, available resources, and practice is lagging. Furthermore, resources and advancements created by recent initiatives, such as 'Mind the Gap', appear not to be communicated to the general public and are not integrated into medical curricula. In this perspective article, we propose that student‐led initiatives hold the potential to close the existing gap in diversity and racial equity in dermatology and share our experience from a large public‐engagement campaign in South West England. We describe how student‐led initiatives allow medical students to advocate for diversity and equity within their institutions while delivering much‐needed education to ethnic minoritiy<g class="gr_ gr_671 gr‐alert gr_spell gr_inline_cards gr_disable_anim_appear ContextualSpelling ins‐del multiReplace" id="671" data‐gr‐id="671">sed</g> communities.

In light of recent advocacy and activism against racism, more attention has been brought to the inequality of healthcare outcomes in ethnic minority populations. The United Kingdom's population is rapidly diversifying, with around 1.9 million (3.4%) identifying as Afro‐Caribbean, 3 million (5.3%) as South Asian, and 1.2 milslion (2.1%) as mixed racial background in 2011 census, with the numbers today higher than ever before. Despite this, self‐identifying as an ethnic minority is correlated with poorer health outcomes.^1^ The racial and ethnic disparity roots are multi‐factorial and stem from poorer access to healthcare, lower levels of health literacy, inequality in research, distrust towards research community, and potential bias amongst clinicians.^2^ This issue is particularly pertinent in dermatology. Multiple studies have shown that unfamiliarity with dermatological presentations in skin of colour (SOC) contributes to underdiagnosis or misdiagnosis of skin cancer and numerous other diseases, including atopic dermatitis, acne, eczema, ringworm, and skin manifestations of autoimmune conditions.^3^ One explanation for this is limited diversity in the depictions of skin diseases in medical education and resources, which are yet to be negated by current efforts. Whilst educating medical professionals and students is key, the wealth of information already available appears inadequately communicated to the public. Addressing this gap will support the general public in recognising skin conditions and seeking appropriate and timely medical attention.

The lack of diversity in educational resources has been addressed by efforts at both the institutional and individual levels.^4^ The British Association of Dermatologists (BAD) recently highlighted the important role of journal editors and publishers in improving healthcare diversity, racial equity, and inclusion.^5^ One aim is to encourage the use of images that reflect variations in disease across all skin tones, representing our diverse patient population. This has also been highlighted by the wider dermatology research community to address underrepresentation of SOC in medical education.^1^ A UK‐based project by the ‘Don't Forget the Bubbles' research team and the Royal London Hospital developed a continually growing repository of paediatric dermatological conditions in SOC.^6^ On an individual level, collaboration between medical professionals, students and their institutions has led to campaigns that have had national impact. A key example is the ‘Mind the Gap' project initiated by Malone Mukwende, a medical student at St. George's University of London, who created a clinical handbook of signs and symptoms in SOC. The project was repeatedly featured in press and on television both in the UK and the US and was perhaps the furthest‐reaching project of its kind.^7^ The reach of this initiative was further amplified by the BAD through publicising its resources. This has led to increased awareness of inequalities in diverse dermatology education amongst clinicians and medical students.^4^

Despite these endeavours, the depiction of dermatology in SOC remains inadequately described and integrated into medical curricula.^8^ Barriers remaining include a dearth of diverse educational resources, lack of diversity within medical faculties, and institutional mindset, which often results in passive and tokenistic contributions to the curriculum.^1^ Current efforts are yet to result in perceptible improvements for the general public. To ensure equal outcomes within healthcare, patients require sufficient education. This should cover the scope of disease prevention, health promotion, and presentation in ethnic minority populations. For clinicians to be able to care for our increasingly diverse society, they must be exposed to diversity and be taught to recognise conditions appropriately, irrespective of skin tone or ethnic background. This change should start during pre‐clinical training and early clinical experiences,^9^ and not solely geared towards practising dermatologists and general practitioners. Raising awareness of the present bias in dermatology, starting from an early stage, will likely improve student confidence and ability to accurately diagnose conditions in SOC.^9^ The potential impact of this paradigm shift will provide benefit beyond dermatology and hopefully encourage students to consider other factors such as religion, culture and health beliefs when interacting with patients.

We propose that bottom‐up change, implemented through student‐led grassroots initiatives, can aid development of educational resources, and disseminate awareness of health inequalities amongst tomorrow's clinicians. In addition, such events bridge the gap between collaborative, multidisciplinary work within the medical field and recipients of care. To exemplify the benefits of student‐led initiatives, we share our experience of holding a large‐scale public engagement event Figure 1. Our project ‘Skin Diversity: Closing the Gap’ was funded and supported by the University of Exeter with the aim to engage medical students to deliver valuable public health education. This initiative was delivered at the Exeter Respect Festival 2022. Dedicated to equality and diversity, the festival has a footfall of around twenty thousand people. The primary aim of our project was to increase public awareness of skin presentations, with particular emphasis on under‐representation in SOC. We also wanted to broaden access to resources on preventing, recognising, and managing common skin conditions. At the heart of our initiative was engaging in personalised discussions with the public on diversity in dermatology and overall skin health.

**FIGURE 1:**
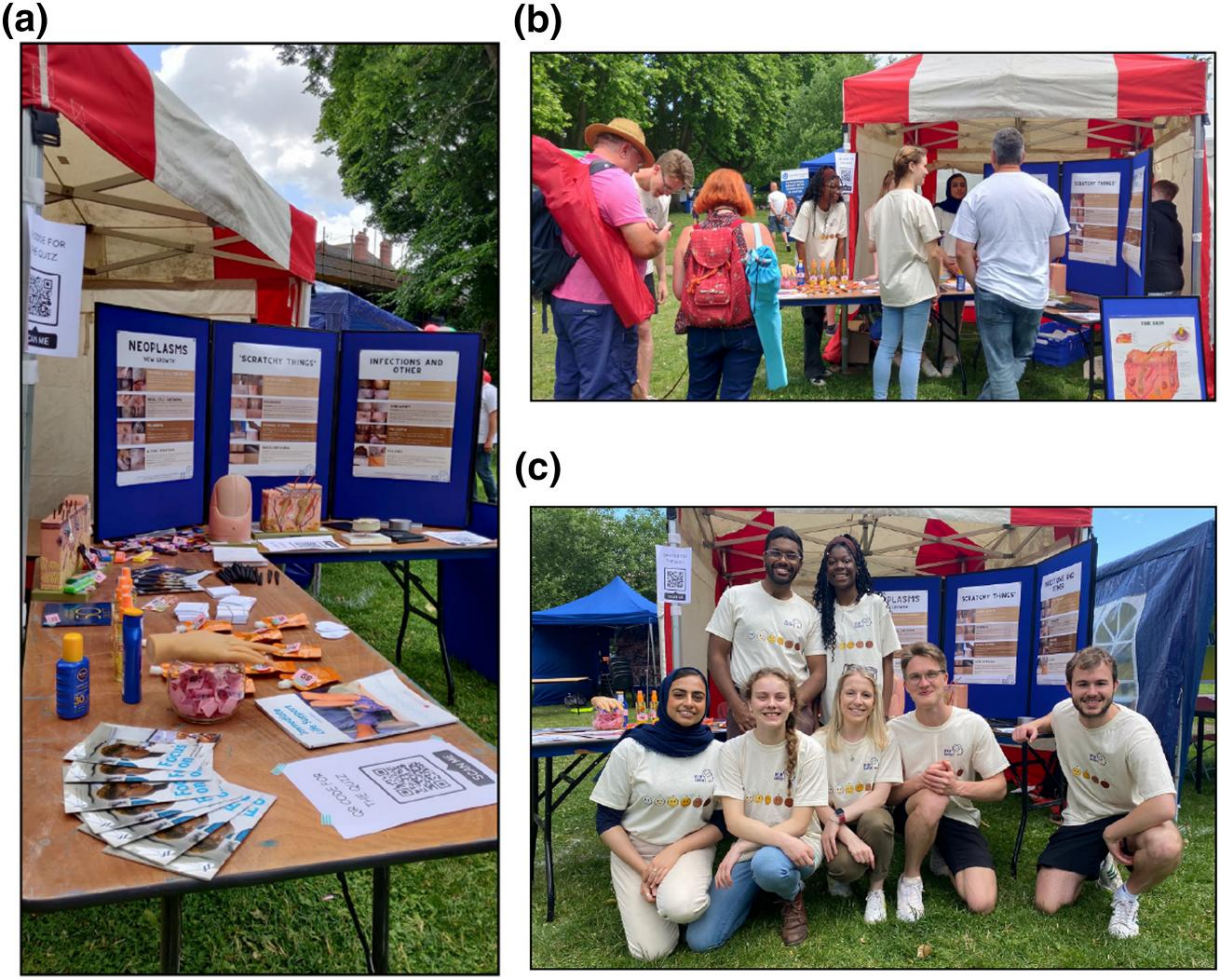
(A–C) illustrate some moments from the event ‘Skin diversity: closing the gap’. Panel A shows the arrangement of our stall, with incentives (sunscreen sachets and bottles, pens, notepads), an anatomical model of the skin and nails, and posters in the background, panel B shows members of the team chatting to members of the public on the day, panel C depicts the seven medical students who organised the project and engaged with the public during the duration of a 2 day festival

We created posters summarising the most common skin conditions (Figure 2) in accessible terms, with details on pathophysiology, management, and prevention. The images included were used with permission by the ‘Mind the Gap’ team and captured from their webpage (https://www.blackandbrownskin.co.uk). Unsurprisingly, the under‐representation of SOC was apparent during our research for appropriate images, as relevant resources were scarce. Inevitably, the minimal evidence‐base has led to a lack of accessible and updated patient resources. This discrepancy was further made evident through the lack of darker skinned models in our medical school's teaching resource collection, and consequently on our stall. Encouragingly, this was raised in feedback following our intervention, suggesting that we successfully increased public discourse around this issue. We initiated discussions by gauging participant experiences of dermatology and encouraged them to pose questions surrounding conditions of interest and areas requiring clarification. Many members of the public were comfortable sharing personal anecdotes on skin health, leading us to engage in a range of conversations tailored to each individual.

FIGURE 2(A–C) show the posters created by our team and used as educational resources during the public engagement event. Poster A discusses skin cancers and pre‐cancerous changes and emphasises the importance of sun protection, poster B shows most common infections on fair and darker skin, poster C depicts most common conditions associated with erythema and irritation of the skin. Digital versions of posters were available through a QR code

**Figure:**
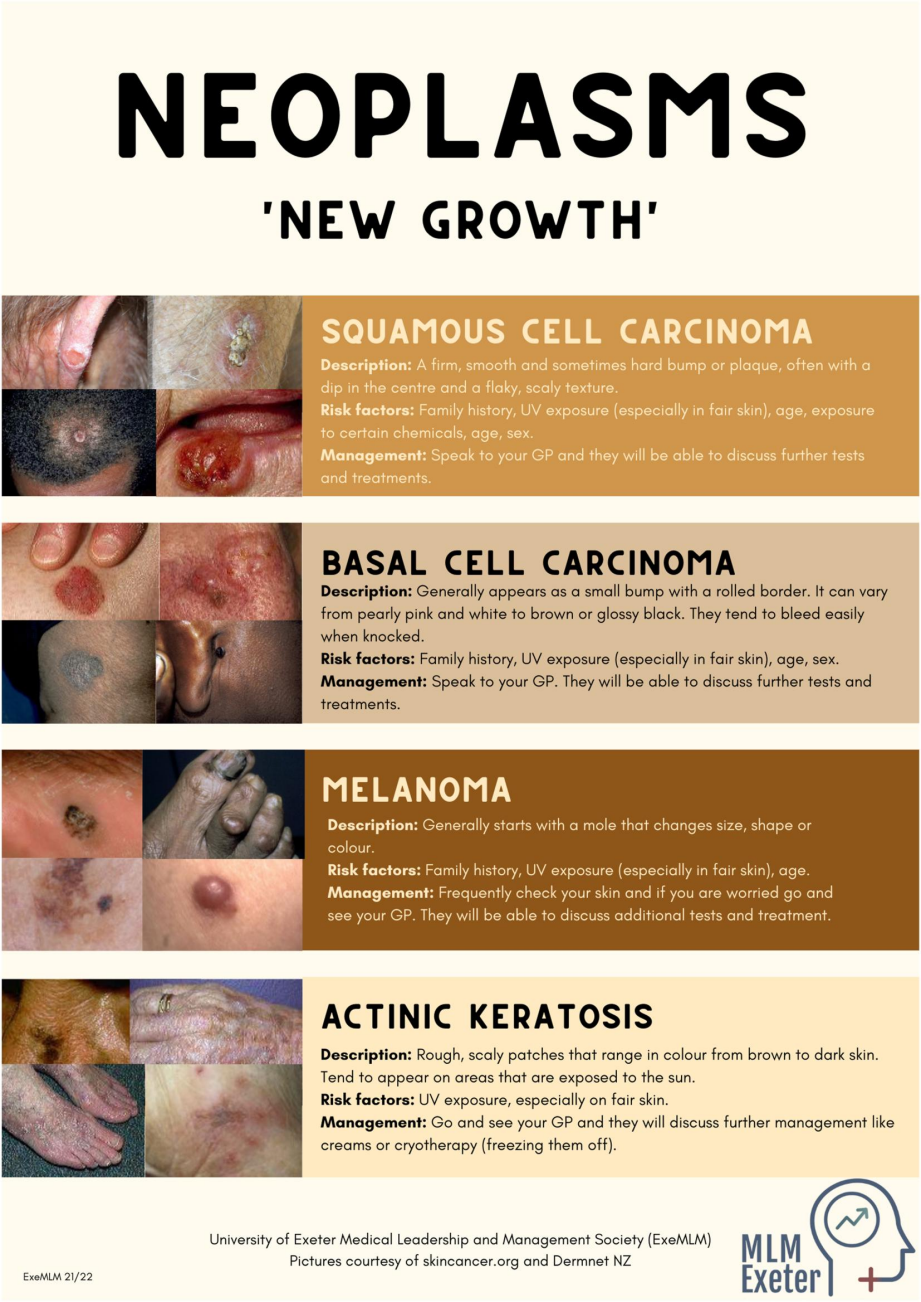

**Figure:**
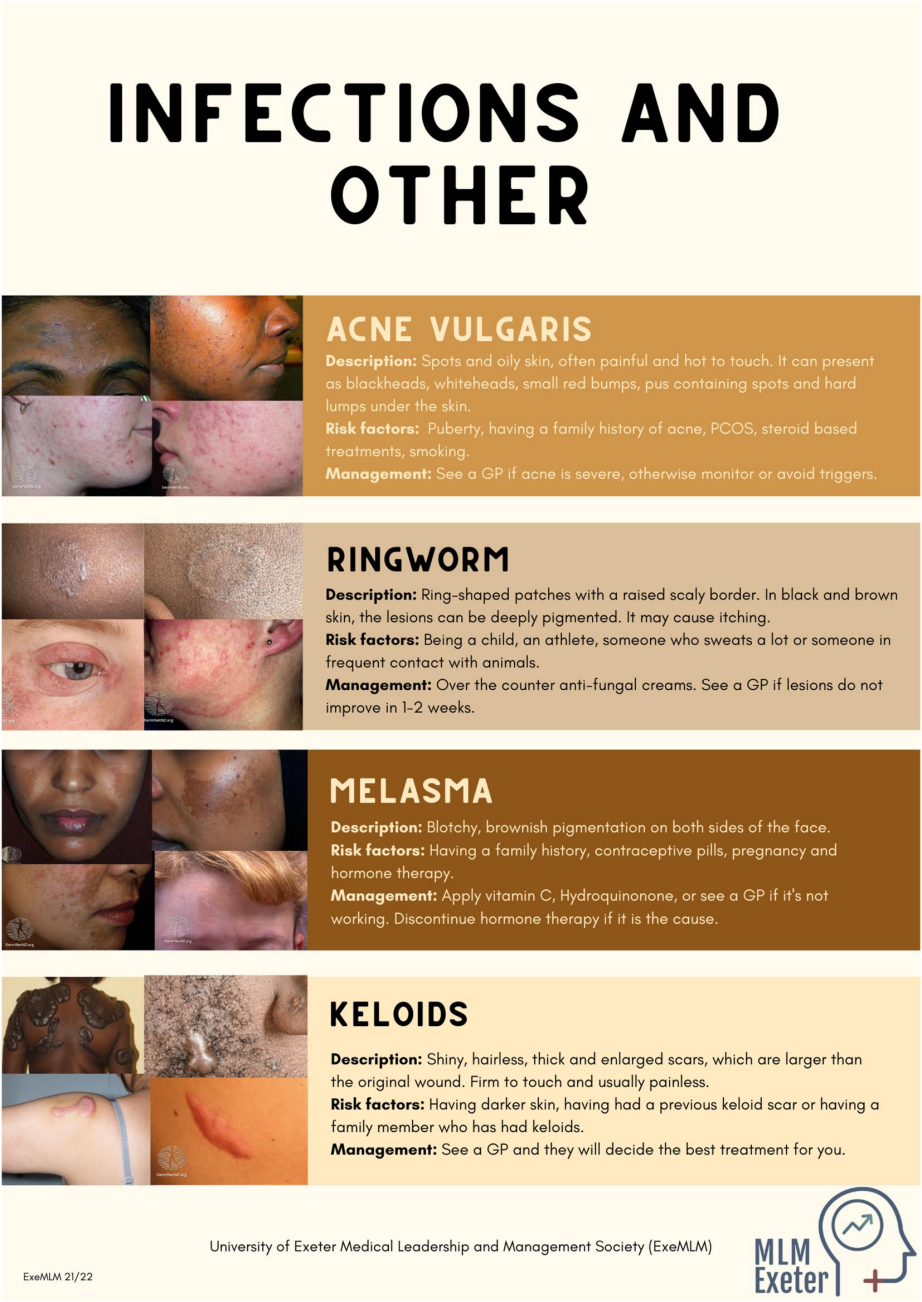

**Figure:**
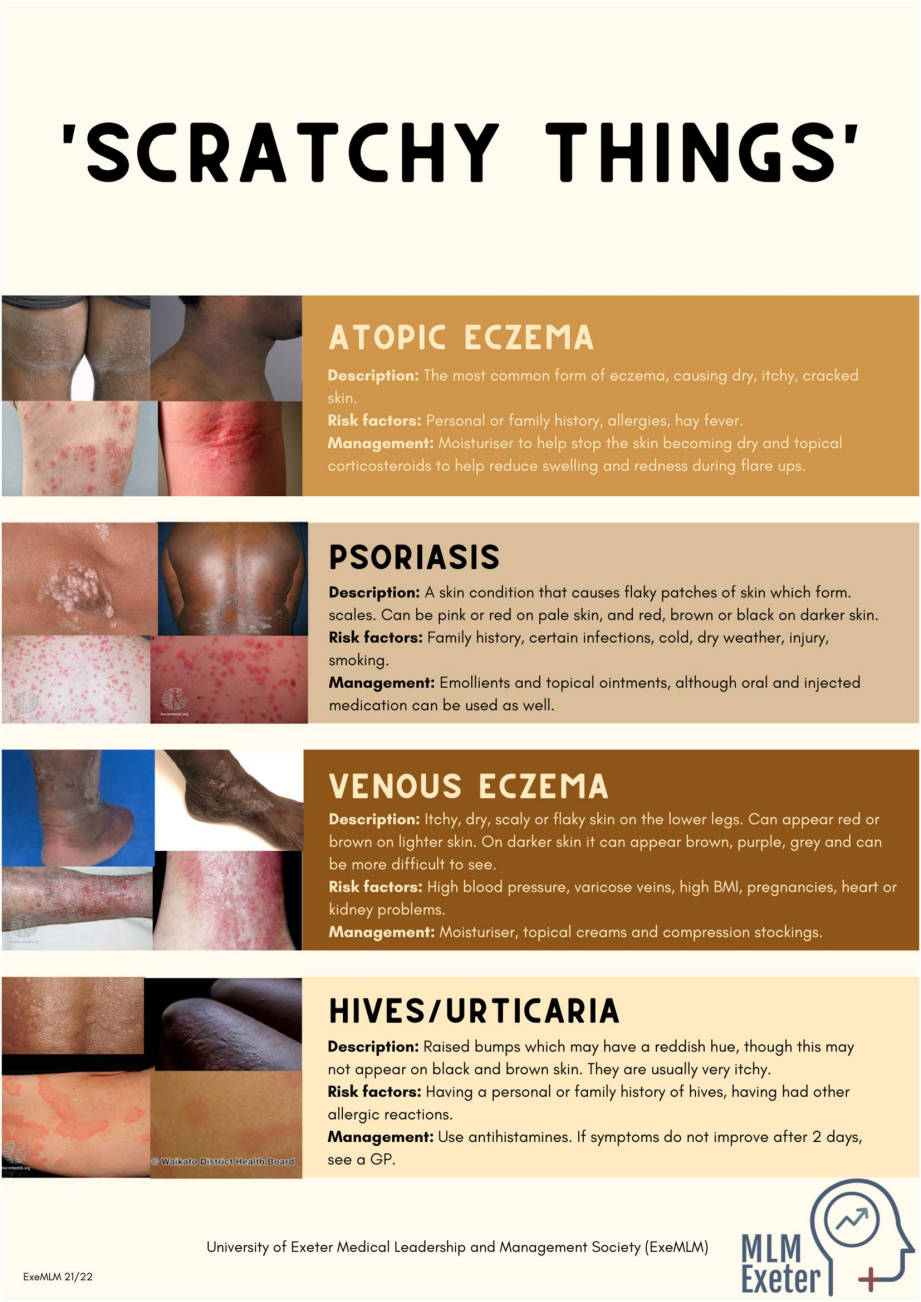

One recurring theme was how certain conditions often result in an ‘angry red rash’, that is, an itchy erythematous rash secondary to infection, hypersensitivity, or drug reactions, which is not apparent on darker skin tones. This is in line with a recent suggestion that the term ‘erythema’ is misused and varies with skin tone, resulting in underrated disease severity in darker skin types.^10^ However, after reviewing the posters, participants were more likely to recognise eczema and ringworm in both light and dark skin tones, despite the absence of reddening. Another key discussion point was the misconceptions surrounding skin cancer. This topic was of particular importance due to the significantly higher rates of skin cancer in the South West England. Although more prevalent in fairer skin, poor cancer recognition and delayed diagnoses result in poorer prognosis in ethnic minorities.^3^ We discussed specific cancer subtypes and the variations in presentations in SOC. Participants were then able to recognise acral lentiginous melanoma beneath the nail as the most worrying of all conditions presented to them.

In total, we spoke to over five hundred members of the public during the 2 day event. The costs of running our project summated only to printing costs and incentives for participation, generously funded by the university's ‘Equality, Diversity and Inclusion’ grant. Evidently, such events present a valuable and financially feasible opportunity for medical students to positively influence public perception of key health issues whilst developing public speaking and complex communication skills. These opportunities enable students to gain early career exposure to how ethnicity influences provision of care, which is an important determinant of patient experience and universal healthcare outcomes.

Medical students can actively collate information from existing resources and campaign for solutions to other disparities within research and education. The publicising of such initiatives also enables medical students to advocate for diversity and racial equality within their institutions. Members of our project voiced their concerns regarding the lack of diversity in our University's teaching resources at a college board meeting. This platform not only facilitated personal and professional development of the students involved, but aided communication between the public and the university. We were also grateful to receive feedback on plans for conducting future initiatives within the medical school and the wider community. Similar positive impacts of student‐led initiatives have been reported by others, including in areas outside of dermatology.^4^

In addition to the benefits received by medical students, such projects also profit the public in several ways. Firstly, they invite individuals to share their experiences and contribute to conversations on socially pertinent issues in medicine in a safe environment to future doctors. It also encourages health promotion in ethnic minority communities and increases accessibility to emerging reliable resources. Such initiatives can effectively communicate current efforts to rectify discrepancies in medical provision and may improve public trust in healthcare professionals.

In conclusion, recent action in addressing health inequalities in dermatology needs to be translated into tangible public benefit, particularly in underrepresented communities. Our grassroots project findings and experiences beyond dermatology strongly suggest that student‐led projects can be indispensable in communicating positive change to the public. If broadly implemented, such initiatives have the potential to catalyse much needed change in both medical education and clinical settings, and ultimately improve downstream patient outcomes.

## AUTHOR CONTRIBUTIONS

**Pavel Loginovic**: Conceptualization (Lead); Funding acquisition (Lead); Methodology (Supporting); Project administration (Lead); Resources (Supporting); Writing – original draft (Lead); Writing – review & editing (Equal). **Natasha Syed**: Conceptualization (Equal); Investigation (Equal); Methodology (Equal); Writing – original draft (Equal); Writing – review & editing (Equal). **Alex M. Parker**: Conceptualization (Equal); Methodology (Equal); Project administration (Supporting); Resources (Lead); Writing – original draft (Equal); Writing – review & editing (Equal). **Nel G. Williams**: Conceptualization (Supporting); Methodology (Supporting); Resources (Supporting); Visualization (Lead); Writing – original draft (Equal); Writing – review & editing (Equal). **Navin M. Nagesh**: Conceptualization (Supporting); Project administration (Supporting); Resources (Supporting); Supervision (Lead); Writing – original draft (Supporting); Writing – review & editing (Lead).

## CONFLICT OF INTEREST

The authors declare that there is no conflict of interest that could be perceived as prejudicing the impartiality of the research reported.

## Data Availability

Data sharing not applicable to this article as no datasets were generated or analysed during the current study.
